# Supplementary material for: Development of a multiplex Loop-Mediated Isothermal Amplification (LAMP) assay for on-site diagnosis of SARS CoV-2
Source: PLoS One. 2021 Mar 3;16(3):e0248042. doi: 10.1371/journal.pone.0248042 (PMC7928493; doi:10.1371/journal.pone.0248042)
Supplement: S1 Fig — Numbers (1–5) indicated diluted samples/μL (1.0–1.0 × 10^−3^ copies/μL) and negative control (distilled water (DW) as non-template control). (DOCX) [file pone.0248042.s001.docx]

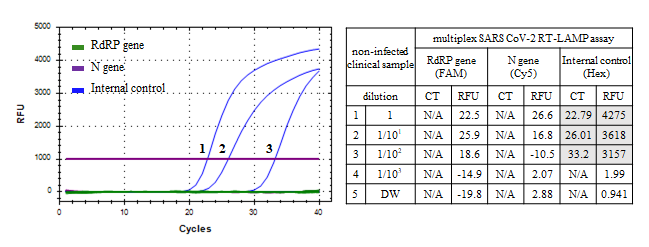
 **S1 Fig. Limit of detection of the multiplex SARS CoV-2 RdRP/N/IC RT-LAMP assay for non-infected clinical samples (ranging from 1 to 10^-3^ copies/μL).** Numbers (1-5) indicated diluted samples/μL (1.0 - 1.0 × 10^-3^ copies/μL) and negative control (distilled water (DW) as non-template control).
