# Supplementary material for: Development of a multiplex Loop-Mediated Isothermal Amplification (LAMP) assay for on-site diagnosis of SARS CoV-2
Source: PLoS One. 2021 Mar 3;16(3):e0248042. doi: 10.1371/journal.pone.0248042 (PMC7928493; doi:10.1371/journal.pone.0248042)
Supplement: Attachment — Submitted filename: Response to Reviewers_SARS CoV-2 v7.docx [file pone.0248042.s002.docx]

Response to Reviewers

# Dear editor,

We would like to thank you for your interest and detailed comments on our manuscript. We really appreciate it. We revised our manuscript according to the reviewer’s comments.

Review report

Title: Development of a Multiplex Loop-Mediated Isothermal Amplification (LAMP) Assay for On-Site Diagnosis of SARS CoV-2

Author: Woong Sik Jang2¶, Da Hye Lim1¶, Jung Yoon1, Ahran Kim1, Minsup Lim2, Jeonghun Nam2, Richard Yanagihara3, Sook-Won Ryu 4, Bo Kyeung Jung5, Nam-Hee Ryoo 6* and Chae Seung Lim1*

Reviewer #1:

1. This manuscript describes (another) development and evaluation of a LAMP assay for Covid19. There are many publications describing such a test (see list below). Consequently the authors should make clear why their test has an add on value.

Loop mediated isothermal amplification (LAMP) assays as a rapid diagnostic for COVID-19. Kashir J, Yaqinuddin A.Med Hypotheses. 2020 Aug;141:109786. doi: 10.1016/j.mehy.2020.109786. Epub 2020 Apr 25.PMID: 32361529 Free PMC article.

Loop-Mediated Isothermal Amplification (LAMP): A Rapid, Sensitive, Specific, and Cost-Effective Point-of-Care Test for Coronaviruses in the Context of COVID-19 Pandemic. Augustine R, Hasan A, Das S, Ahmed R, Mori Y, Notomi T, Kevadiya BD, Thakor AS.Biology (Basel). 2020 Jul 22;9(8):182. doi: 10.3390/biology9080182.PMID: 32707972 Free PMC article.

A Rapid, Simple, Inexpensive, and Mobile Colorimetric Assay COVID-19-LAMP for Mass On-Site Screening of COVID-19. Chow FW, Chan TT, Tam AR, Zhao S, Yao W, Fung J, Cheng FK, Lo GC, Chu S, Aw-Yong KL, Tang JY, Tsang CC, Luk HK, Wong AC, Li KS, Zhu L, He Z, Tam EWT, Chung TW, Wong SCY, Que TL, Fung KS, Lung DC, Wu AK, Hung IF, Woo PC, Lau SK.Int J Mol Sci. 2020 Jul 29;21(15):5380. doi: 10.3390/ijms21155380.PMID: 32751106 Free PMC article.

Rapid Detection of COVID-19 Coronavirus Using a Reverse Transcriptional Loop-Mediated Isothermal Amplification (RT-LAMP) Diagnostic Platform. Yu L, Wu S, Hao X, Dong X, Mao L, Pelechano V, Chen WH, Yin X.Clin Chem. 2020 Jul 1;66(7):975-977. doi: 10.1093/clinchem/hvaa102.PMID: 32315390 Free PMC article.

RT-LAMP for rapid diagnosis of coronavirus SARS-CoV-2. Huang WE, Lim B, Hsu CC, Xiong D, Wu W, Yu Y, Jia H, Wang Y, Zeng Y, Ji M, Chang H, Zhang X, Wang H, Cui Z.Microb Biotechnol. 2020 Jul;13(4):950-961. doi: 10.1111/1751-7915.13586. Epub 2020 Apr 25.PMID: 32333644 Free PMC article.

Use of the variplex™ SARS-CoV-2 RT-LAMP as a rapid molecular assay to complement RT-PCR for COVID-19 diagnosis. Rödel J, Egerer R, Suleyman A, Sommer-Schmid B, Baier M, Henke A, Edel B, Löffler B.J Clin Virol. 2020 Nov;132:104616. doi: 10.1016/j.jcv.2020.104616. Epub 2020 Aug 31.PMID: 32891938 Free PMC article.

**🡪 As you mention above, all case of SARS CoV-2 RT-LAMP assay showed good performance. However, they used one reaction mixture in one tubes for detecting one gene of SARS CoV-2. Thus, for detecting two gene of SARS CoV-2 and internal control gene in order to increase sensitivities, they need at least three reaction tubes. Furthermore, it is known that LAMP assay is easy to contaminate. So, our multiplex LAMP assay in one tube have advantages for reducing test time and risk of contamination. These contents were included in discussion following as:**

**“Currently, several SARS CoV-2 LAMP primer sets were reported [23-26]. They were mostly developed with fast colorimetric detection of one or two genes suitable for on-site diagnosis [27-30]. However, it has disadvantages in not producing diagnose with multiplex testing and having to test each primer set individually. In particular, the LAMP assay has been reported to be highly susceptible to contamination [31, 32], and the recently reported SARS CoV-2 RT-LAMP assay has also pointed out such a problem [33]. Therefore, if an RT-LAMP test for one clinical sample is performed with three or four LAMP primer sets (including internal control) individually, the degree of contamination may also increase. In addition, when conducting clinical tests in large quantities, the number of clinical trials more than doubles, and the advantage of a rapid diagnosis of the LAMP assay may be diluted. Therefore, the multiplex SARS CoV-2 RdRP/N/IC RT-LAMP assay developed in this study has an advantage in minimizing the contamination and enabling a mass diagnosis.” (line 263-274)**

2. Although the work is well designed and presented, a concern is that the sample size is rather limited. A sample size calculation is not provided. This must be done.

**🡪 We added sentence based on the comment following as:**

**“To estimate the number of samples required for clinical test of the multiplex RT-LAMP assay, the following formula was used:**

$$\boldsymbol{n}\boldsymbol{\geq}\frac{\mathbf{(1.96)}^{\mathbf{2}}\boldsymbol{p}\mathbf{(1-}\boldsymbol{p}\mathbf{)}}{\boldsymbol{x}^{\mathbf{2}}}$$

**where p is the suspected sensitivity, and x is the desired margin of error [15, 16]. The true-positive rate (sensitivity) was defined as the proportion of SARS-CoV-2 positive which is correctly identified by the multiplex RT-LAMP assay compared to the AllplexTM 2019-nCoV Assay (Seegene, Inc., Seoul, South Korea). We suspected the sensitivity and specificity of the multiplex RT-LAMP assay to be 95% with a desired margin of error of 0.04%. Under these conditions, the number of required samples is 114.0475 (rounded up to 115) per group. In this experiment, we have tested total 292 samples (130 positive and 162 negative).” (Line 92-101)**

3. The paper would stand out amongst all other papers in this particular field if they would have assesses a much larger sample size. This is not the case. Therefore, I recommend that the authors should extend their study population before re-submitting the manuscript.

**🡪 As your suggestion, we assess the multiplex LAMP assay with 293 clinical samples (130 SARS CoV-2 samples and 162 negative samples.**

4. A non-template control should be included in the assay.

**🡪 We have added the non-template control in Figure 1 Legend based on the comment following as:**

**“Numbers (1-10) indicated plasmid copy numbers/μL (1.0 × 10^8^ - 1.0 × 10^0^ copies/μL) and negative control (distilled water (DW) as non-template control).” (Line 474-475)**

5. The resolution of figure 2 is rather low.

**🡪 We have corrected the resolution of figure 2 based on the comment.**
